# Anticoagulant Therapy Is Associated With Decreased Long-Term Mortality in Splenic Infarction Patients: A Multicenter Study

**DOI:** 10.3389/fmed.2021.778198

**Published:** 2021-11-29

**Authors:** Chieh-Ching Yen, Chih-Kai Wang, Chung-Hsien Chaou, Shou-Yen Chen, Jhe-Ping Lin, Chip-Jin Ng

**Affiliations:** ^1^Department of Emergency Medicine, Chang Gung Memorial Hospital, Taoyuan, Taiwan; ^2^College of Medicine, National Yang Ming University, Taipei, Taiwan; ^3^College of Medicine, Chang Gung University, Taoyuan, Taiwan; ^4^Chang Gung Medical Education Research Center, Taoyuan, Taiwan

**Keywords:** anticoagulant, splenic infarction, mortality, outcome, treatment

## Abstract

**Background:** Patients with splenic infarction (SI) are associated with a prothrombotic state and are vulnerable to subsequent thromboembolic complications. However, due to its rarity, there is no established treatment modality in this population. We aimed to examine the effect of anticoagulant therapy in SI patients.

**Methods:** We performed a multicenter retrospective cohort study of 86 SI patients. Patients were categorized as anticoagulant users and anticoagulant non-users. The associations between anticoagulant therapy, all-cause mortality, thromboembolic events and bleeding events were evaluated.

**Results:** Forty-five patients (52.3%) received anticoagulant therapy during the follow-up periods. The all-cause mortality rate was 6.86 per 100 patient-years. Anticoagulant therapy was associated with 94% improved survival (HR = 0.06; Cl 0.007–0.48; *p* = 0.008), while the risk factors for all-cause mortality were prior stroke (HR = 13.15; Cl 2.39–72.27; *p* = 0.003) and liver cirrhosis (HR = 8.71; Cl 1.29–59.01; *p* = 0.027). Patients with anticoagulant therapy had a higher event-free survival curve for thromboembolic complications (*p* = 0.03) but did not achieve a significant difference after adjustment using the Cox regression model as a time-dependent covariate (HR = 0.57; Cl 0.13–2.45; *p* = 0.446). There was no significant difference in the risk of bleeding events between the groups (*p* = 0.728).

**Conclusions:** Anticoagulant therapy in patients with SI was associated with better survival and was not related to an increased bleeding risk.

## Introduction

Splenic infarction (SI) is an uncommon diagnosis in the general population. It accounts for 0.016% of admissions to an academic general hospital (1). The splenic artery and its branches constitute the main blood supply of the spleen. SI may develop upon occlusion of these vessels owing to several predisposing factors associated with intrasplenic thrombosis, arteriosclerosis, and embolism (2). The underlying etiologies include cardiogenic emboli in which atrial fibrillation is most common, autoimmune disease, infection, hematologic disease, and malignancy (1). Most patients present with abdominal pain, which is often localized to the left upper quadrant (3). Laboratory investigations are less helpful, although some series reported leukocytosis and mildly elevated LDH levels in these patients (3, 4). Contrast-enhanced computed tomography (CT) is currently the first-line imaging study to diagnose SI. It allows accurate detection of infarcted viscera and other urgent conditions in patients with acute abdominal pain (5).

The use of anticoagulant therapy in SI patients aims to achieve vessel recanalization and decrease mortality by preventing subsequent thromboembolic complications. However, patients taking anticoagulants pose a higher bleeding risk, and the management of bleeding complications in patients under anticoagulant therapy is challenging (6). Previous studies demonstrated that 30% to 100% of patients with SI received anticoagulant therapy (1, 3, 7). To date, no studies have directly compared patients who initiated anticoagulants to those without treatment in the future risk of thromboembolic events and long-term overall survival. The impact of anticoagulant therapy on outcomes in SI patients remains under speculation. Accordingly, to address the gap in the literature, this study was designed to examine the association between anticoagulant therapy, thromboembolic complications, all-cause mortality, and bleeding events in patients with SI.

## Materials and Methods

### Study Design and Setting

After receiving approval from the Chang Gung Medical Foundation Institutional Review Board (IRB no. 202101187B0), all adult patients who met the inclusion criteria in the study from January 1, 2005, to June 1, 2021, were retrospectively enrolled for analysis. The study sites were six hospitals in Taiwan that used the same electronic medical records (EMR) system, including two tertiary medical centers, three regional hospitals, and one district hospital. The combined capacity of the study sites was a total of over 9,000 beds and an annual emergency department (ED) visit count of 500,000 patients. This study was carried out in accordance with The Strengthening the Reporting of Observational studies in Epidemiology (STROBE) guidelines.

### Patient Selection and Data Collection

We first identified all adult patients with International Classification of Diseases (ICD)-9 code 289.59 and ICD-10 code D735 of splenic infarction (SI) who presented to the ED during the study period. The patients selected by EMR were reviewed by two emergency physicians (CCY and CKW). To reduce the heterogeneity of the study population, the exclusion criteria were patients with active non-hematologic malignancy, hematologic disease, and infective endocarditis. Patients under 18 years of age at discharge, incomplete medical records, or the protected population due to original IRB approval were also excluded.

Demographic information and previous medical history, including hypertension, diabetes mellitus, coronary artery disease, congestive heart failure, atrial fibrillation, chronic kidney disease, prior venous thromboembolism, prior stroke, and liver cirrhosis, were collected. Similarly, prior anticoagulant or antiplatelet therapy was also retrieved. Initial vital signs and clinical symptoms, including blood pressure, heart rate, abdominal pain, back pain, nausea or vomiting, dyspnea, and fever, were obtained. Laboratory findings on initial presentation included white cell count, hemoglobin, platelet count, international normalized ratio, creatinine, and aspartate aminotransferase. All images were reviewed, and a splenic infarct on computed tomography was defined as a peripheral wedge-shaped hypodense region. The presence of splenomegaly (defined as a spleen measuring > 12 cm in any plane), single infarct, and multiple infarcts were further evaluated.

Anticoagulant therapy was defined as intravenous heparin administration followed by low molecular weight heparin and then oral anticoagulants, low molecular weight heparin administration followed by oral anticoagulants, or oral anticoagulants alone. Oral anticoagulants included vitamin K antagonists and direct oral anticoagulants.

### Measurable Outcomes

The primary outcomes were recurrent thromboembolic events, bleeding events, and all-cause mortality. Thromboembolic events included ischemic stroke, acute coronary syndrome, acute limb ischemia, recurrent SI, and thrombosis in the visceral arteries or veins. Bleeding events included intracranial hemorrhage, gastrointestinal bleeding, internal bleeding, vaginal bleeding, and hematuria.

### Statistical Analysis

Patient characteristics, presentations, laboratory findings, imaging findings, and clinical outcomes were reported as numbers (percentages) for categorical variables and means (standard deviations, SDs) for continuous variables. Comparison of the two groups between patients with or without anticoagulant therapy involved chi-square tests or Fisher's exact test, as appropriate, for categorical variables, independent Student's t-tests for normally distributed continuous variables and Mann–Whitney U-tests for skewed continuous variables. Kaplan-Meier analyses were performed to assess time-to-event data for thromboembolic events and all-cause mortality between the two groups, and the log-rank test was used to determine any statistically significant difference. To identify independent predictors of thromboembolic events and all-cause mortality, we used a stepwise approach to select variables with *p* <0.10 in univariate analysis to enter the final multivariate Cox proportional hazards model. Anticoagulant therapy was included as a time-dependent variable based on a time-varying effect and to minimize the risk of immortal time bias (8, 9). All analyses were performed using SPSS software v20.0 (SPSS Inc., Chicago, IL). A two-sided *p* < 0.05 was considered statistically significant.

## Results

### Patient Characteristics

**Figure 1:**
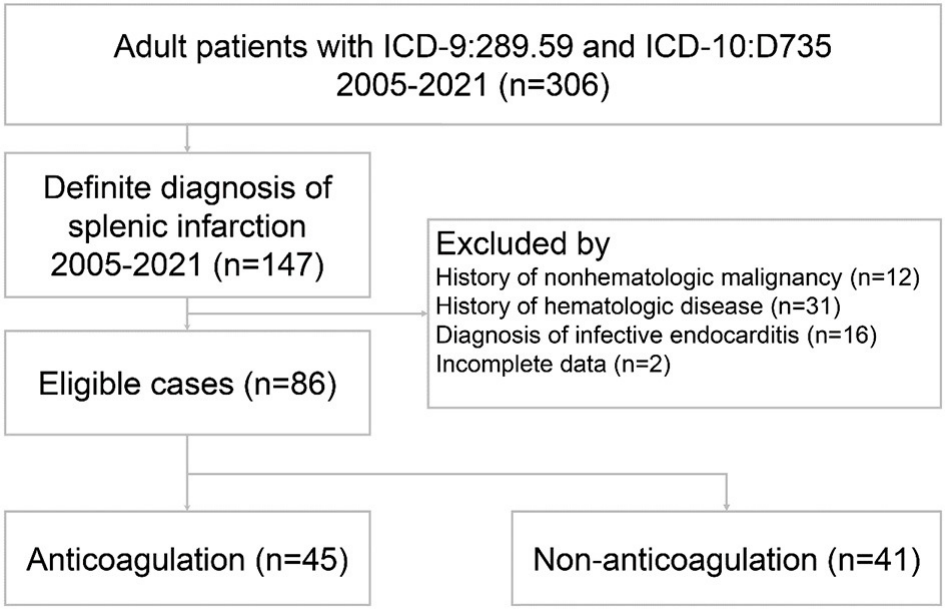
Flow chart of patient selection.

During the study period, 306 patients were discharged with the diagnosis of ICD-9: Other diseases of spleen (289.59) or ICD-10: Infarction of spleen (D735). Since that no specific coding in ICD-9 for splenic infarction and the coding: Other diseases of spleen (289.59) included the splenic disorders other than splenic infarction, 159 patients were excluded due to a lack of a definite diagnosis of splenic infarction. Sixty-one patients were subsequently excluded owing to concomitant non-hematologic malignancy, hematologic disease, infective endocarditis, and incomplete data. Finally, a total of 86 patients met the entry criteria for the study (Figure 1).

**Figure 2:**
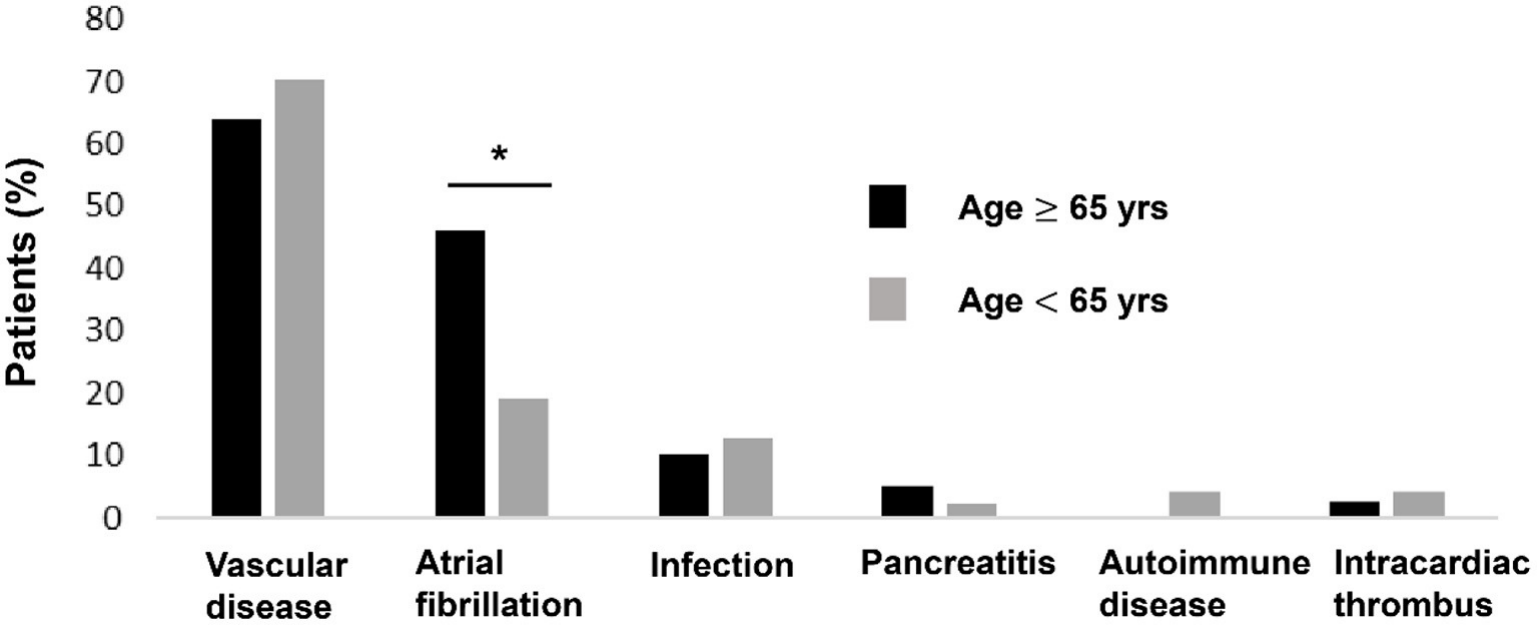
Distribution of SI etiologies stratified by patient age. *P < 0.05.

All patients were diagnosed with SI by contrast-enhanced CT. Vascular diseases were the leading etiologies (*N* = 58, 67%), which consisted of hypertension (*N* = 46), splenic artery thrombosis (*N* = 4), splenic artery dissection (*N* = 1), splenic vein thrombosis (*N* = 2), celiac artery thrombosis (*N* = 3) and celiac artery dissection (*N* = 3), followed by atrial fibrillation (*N* = 27, 31%), infection (*N* = 11, 13%), intracardiac thrombus (*N* = 3, 3%), acute pancreatitis (*N* = 3, 3%), and autoimmune disease (*N* = 2, 2%). Seventeen of the patients (20%) had more than one plausible predisposing factor. Examples included combinations of hypertension plus atrial fibrillation, infection plus atrial fibrillation, and hypertension plus splenic artery thrombosis. Eleven of the patients (13%) had no recognized morbidity predisposing to SI. The various types of etiologies were stratified by patient age: young (age <65 years) and old (age ≥65 years) patients (Figure 2). The proportion of atrial fibrillation was significantly higher in the old patients (46 vs. 19%, *p* = 0.007).

Forty-five patients (52.3%) received anticoagulant therapy, while forty-one (47.7%) patients did not. Among 45 patients with anticoagulant therapy, 15 received intravenous heparin followed by low molecular weight heparin and then oral anticoagulants (12 had vitamin K antagonists, and 3 had direct oral anticoagulants, including 2 edoxaban and 1 apixaban), 14 received low molecular weight heparin followed by oral anticoagulants (7 had vitamin K antagonists, and 7 had direct oral anticoagulants, including 2 dabigatran, 2 rivaroxaban, 2 apixaban and 1 edoxaban), and 16 were directly prescribed oral anticoagulants (10 had vitamin K antagonists, and 6 had direct oral anticoagulants, including 4 rivaroxaban and 2 apixaban).

**Figure 3:**
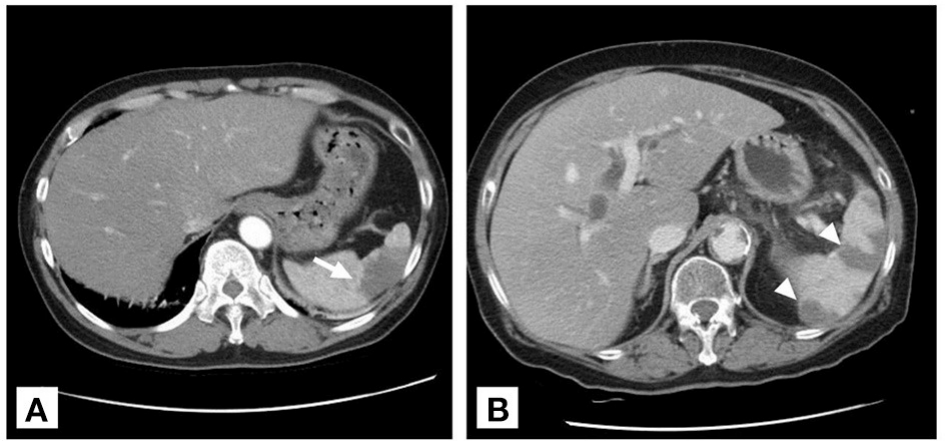
Two patterns of splenic infarction on contrast-enhanced CT. **(A)** Wedge infarct (arrow) in a 49-year-old male with celiac trunk dissection. **(B)** Multiple infarcts (arrowhead) in an 80-year-old woman with acute pancreatitis.

The baseline characteristics of the study population are shown in Table 1. Patients with anticoagulant therapy were non-significantly younger than those without anticoagulant therapy (58 vs. 64.2 years, *p* = 0.075). Regarding initial vital signs in the ED, diastolic blood pressure was significantly higher in the anticoagulation group (88.4 vs. 80.6 mmHg, *p* = 0.045), while fever, systolic blood pressure, and heart rate did not significantly differ between the two groups. There were non-significantly more comorbidities of atrial fibrillation (40 vs. 22%, *p* = 0.072) and significantly fewer comorbidities of liver cirrhosis (2.2 vs. 22%, *p* = 0.006) in patients prescribed anticoagulants. Of all patients with a diagnosis of SI, the main symptoms were left upper quadrant or left flank pain (64%), followed by nausea or vomiting (22.1%), abdominal pain at other sites (17.4%), back pain (15.1%), fever (10.5%), and dyspnea (9.3%). There was no significant difference in the initial presentation between patients with or without anticoagulant therapy. Among patients undergoing laboratory investigations, hemoglobin levels were higher in anticoagulant users than in anticoagulant non-users (14.2 vs. 12.0 g/dL, *p* = 0.001) (Table 2). No significant differences in CT findings of single SI, multiple SIs, and splenomegaly were observed between the two groups (Figure 3).

**Table 1:** Patient characteristics according to anticoagulant therapy.

**Variable**	**Anticoagulation (*N* = 45)**	**Non-anticoagulation (*N* = 41)**	***p***
Age (year)	58.0 ± 14.5	64.2 ± 17.3	0.075
Male	31 (68.9)	24 (58.5)	0.318
Prior anticoagulant use	6 (13.3)	1 (2.4)	0.112
Prior antiplatelet use	10 (22.2)	6 (14.6)	0.366
Concomitant antiplatelet use	7 (15.6)	7 (17.1)	0.849
Systolic blood pressure (mmHg)	145.7 ± 26.9	147.3 ± 34.2	0.808
Diastolic blood pressure (mmHg)	88.4 ± 15.2	80.6 ± 20.1	0.045
Heart rate (beats/min)	89.9 ± 24.1	91.0 ± 21.6	0.838
Smoking history	19 (42.2)	12 (29.3)	0.211
**Previous medical history**			
Hypertension	23 (51.1)	23 (56.1)	0.643
Diabetes mellitus	9 (20.0)	12 (29.3)	0.318
Coronary artery disease	8 (17.8)	10 (24.4)	0.452
Congestive heart failure	8 (17.8)	5 (12.2)	0.470
Atrial fibrillation	18 (40)	9 (22)	0.072
Chronic kidney disease	6 (13.3)	7 (17.1)	0.629
Prior VTE	3 (6.7)	0 (0)	0.243
Prior stroke	6 (13.3)	4 (9.8)	0.741
Liver cirrhosis	1 (2.2)	9 (22)	0.006

*Count data are expressed as number (percentage) and continuous values are expressed as mean ± SD*.

*VTE, venous thromboembolism*.

**Table 2:** Clinical presentations, laboratory exams, and CT findings of patients with splenic infarction according to anticoagulant therapy.

**Variable**	**Anticoagulation**	**Non-anticoagulation**	***p***
	**(*N* = 45)**	**(*N* = 41)**	
**Initial presentation**			
LUQ/left flank pain	31 (68.9)	24 (58.5)	0.318
Abdominal pain^†^	10 (22.2)	5 (12.2)	0.221
Back pain	7 (15.6)	6 (14.6)	0.905
Nausea/Vomiting	10 (22.2)	9 (22.0)	0.976
Dyspnea	7 (15.6)	1 (2.4)	0.060
Fever	4 (8.9)	5 (12.2)	0.731
**Laboratory exam**			
White cell count (10^3^/uL) *n* = 77	11.4 ± 4.1	15.8 ± 15.2	0.077
Hemoglobin (g/dL) *n* = 77	14.2 ± 2.6	12.0 ± 2.9	0.001
Platelet (10^3^/uL) *n* = 77	243.8 ± 114.0	193.7 ± 109.8	0.055
INR *n* = 64	1.2 ± 0.4	1.2 ± 0.2	0.380
Creatinine (mg/dL) *n* = 68	1.0 ± 0.4	1.0 ± 0.3	0.932
AST (U/L) *n* = 28	54.7 ± 54.8	46.8 ± 46.2	0.698
**CT findings**			
Single infarction	31 (68.9)	27 (65.9)	0.764
Multiple infarction	14 (31.1)	14 (34.1)	0.764
Splenomegaly	5 (11.1)	8 (19.5)	0.277

*Count data are expressed as number (percentage) and continuous values are expressed as mean ± SD*.

*LUQ, left upper quadrant; INR, international normalized ratio; AST, aspartate aminotransferase*.

† *Not localized to left upper quadrant*.

### All-Cause Mortality and Survival Analysis

**Figure 4:**
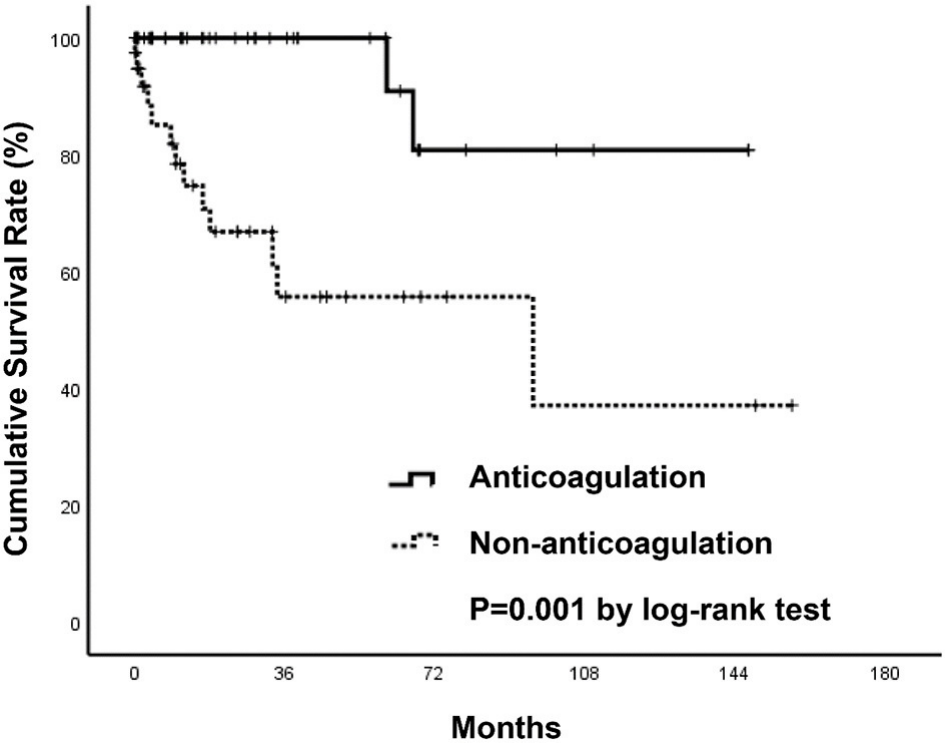
Kaplan-Meier survival curves of patients with all-cause mortality.

During a mean follow-up period of 31 months after a diagnosis of SI, there were two deaths in patients who were anticoagulated (one due to hip fracture followed by sepsis, one due to out-of-hospital cardiac arrest) (1.57 per 100 patient-years) and 13 deaths in patients without anticoagulant therapy (five due to sepsis, four due to ischemic stroke, one due to acute decompensated heart failure, one due to traumatic intracranial hemorrhage, one due to acute chronic liver failure, one due to gastrointestinal bleeding) (14.25 per 100 patient-years) (Table 3). The anticoagulant group showed a significantly higher survival curve than the non-anticoagulant group by Kaplan-Meier analysis (*p* = 0.001) (Figure 4). Univariate and multivariate Cox regression analyses were employed to identify the independent risk factors associated with all-cause mortality. Univariate risk factors included age (HR = 1.04; Cl 1.005–1.08; *p* = 0.025), liver cirrhosis (HR = 9.08; Cl 3.04–27.17; *p* = < 0.001), and splenomegaly (HR = 5.94; Cl 1.98–17.83; *p* = 0.001), while protective factors included male sex (HR = 0.3; Cl 1.10–0.89; *p* = 0.030) and anticoagulant therapy (HR = 0.07; Cl 0.01–0.49; *p* = 0.007). After adjustment, multivariate risk factors for all-cause mortality were prior stroke (HR = 13.15; Cl 2.39–72.27; *p* = 0.003) and liver cirrhosis (HR = 8.71; Cl 1.29–59.01; *p* = 0.027), while anticoagulant therapy was the only independent protective factor (HR = 0.06; Cl 0.007–0.48; *p* = 0.008) (Table 4).

**Table 3:** Primary outcomes of splenic infarction patients with or without anticoagulation at final follow-up.

**Variable**	**Anticoagulation**	**Non-anticoagulation**	***p***
	***N* = 45**	***N* = 41**	
Thromboembolic event	3 (6.7)	8 (19.5)	0.075
Bleeding event	9 (20)	7 (17.1)	0.728
All-cause mortality	2 (4.4)	13 (31.7)	0.001

*Count data are expressed as number (percentage) and continuous values are expressed as mean ± SD*.

**Table 4:** Univariate and multivariate analyses of risk factors for all-cause mortality with Cox proportional hazards model.

	**Univariate**		**Multivariate**	
	**HR (95% CI)**	***p***	**HR (95% CI)**	***p***
Age	1.04 (1.005, 1.08)	0.025	1.04 (0.99, 1.08)	0.099
Male	0.30 (0.10, 0.89)	0.030	0.38 (0.11, 1.32)	0.128
Smoking history	0.83 (0.28, 2.45)	0.740		
Hypertension	0.78 (0.28, 2.17)	0.634		
Diabetes mellitus	1.24 (0.42, 3.65)	0.691		
Coronary artery disease	1.11 (0.31, 4.00)	0.868		
Congestive heart failure	0.51 (0.06, 3.84)	0.509		
Atrial fibrillation	0.92 (0.31, 2.71)	0.882		
Chronic kidney disease	2.22 (0.68, 7.27)	0.189		
Prior stroke	3.50 (0.93, 13.18)	0.064	13.15 (2.39, 72.27)	0.003^*^
Liver cirrhosis	9.08 (3.04, 27.17)	<0.001	8.71 (1.29, 59.01)	0.027^*^
Multiple splenic infarction	0.79 (0.25, 2.51)	0.695		
Splenomegaly	5.94 (1.98, 17.83)	0.001	1.23 (0.22, 7.05)	0.813
Anticoagulant therapy^†^	0.07 (0.01, 0.49)	0.007	0.06 (0.007, 0.48)	0.008^*^

*HR, hazard ratio; 95% CI, 95% confidence interval*.

† *Anticoagulant therapy was analyzed as a time-dependent covariate by time-dependent Cox regression model*.

* *p < 0.05*.

### Thromboembolic Events and Survival Analysis

**Figure 5:**
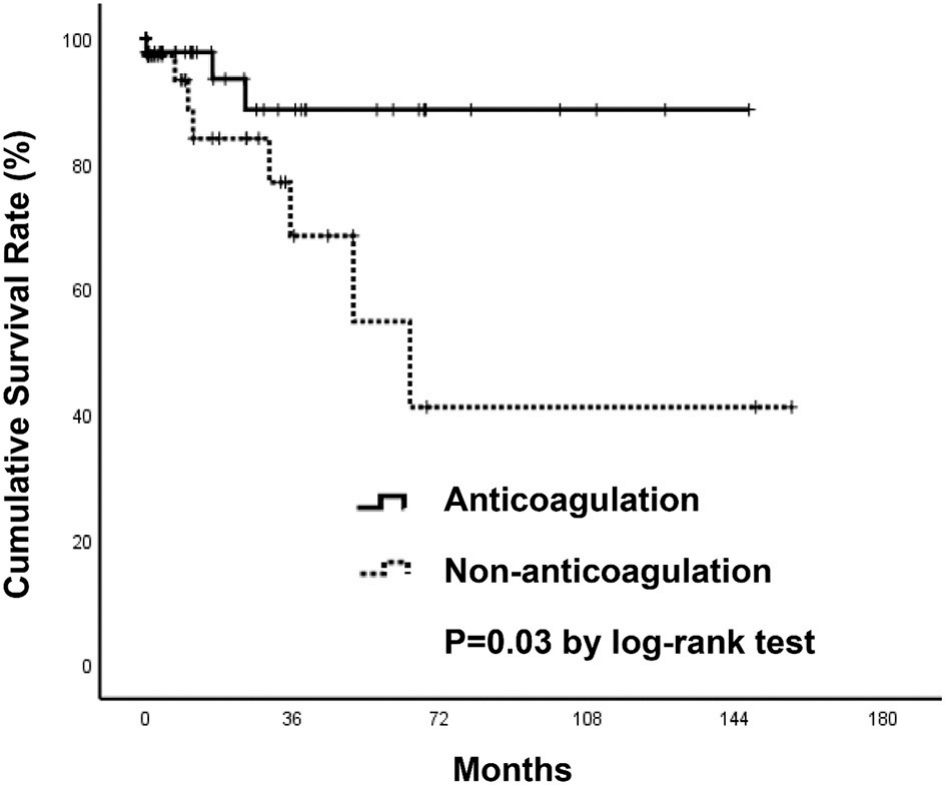
Kaplan-Meier survival curves of patients with thromboembolic events.

Three thromboembolic events (6.7%) occurred in patients who received anticoagulants (two due to ischemic stroke, one to acute limb ischemia), while eight (19.5%) occurred in those without anticoagulant therapy (four due to ischemic stroke, one to recurrent SI with concomitant bilateral renal infarctions, one to recurrent SI, one to acute coronary syndrome, one to septic emboli) during the follow-up period (Table 3). Event-free survival by Kaplan-Meier analysis was significantly higher in the anticoagulation group (*p* = 0.03) (Figure 5). However, after using a Cox regression model with a time-dependent covariate, univariate analysis demonstrated that patients receiving anticoagulant therapy were not associated with fewer thromboembolic events (HR = 0.57; Cl 0.13–2.45; *p* = 0.446). After multivariate analysis, both anticoagulant therapy (HR = 0.71; Cl 0.16–3.09; *p* = 0.707) and atrial fibrillation (HR = 0.30; Cl 0.04–2.48; *p* = 0.262) were not significantly associated with thromboembolic risks (Table 5).

**Table 5:** Univariate and multivariate analyses of risk factors for thromboembolic complications with Cox proportional hazards model.

	**Univariate**		**Multivariate**	
	**HR (95%CI)**	***p***	**HR (95%CI)**	***p***
Age	1.02 (0.98, 1.06)	0.369		
Male	1.61 (0.43, 6.09)	0.481		
Smoking history	1.01 (0.29, 3.45)	0.990		
Hypertension	0.66 (0.20, 2.18)	0.499		
Diabetes mellitus	0.22 (0.03, 1.69)	0.144		
Coronary artery disease	1.49 (0.39, 5.61)	0.559		
Congestive heart failure	1.68 (0.36, 7.82)	0.508		
Atrial fibrillation	0.17 (0.02, 1.31)	0.088	0.30 (0.04, 2.48)	0.262
Chronic kidney disease	2.36 (0.59, 9.44)	0.225		
Prior stroke	1.29 (0.16, 10.32)	0.808		
Liver cirrhosis	1.24 (0.16, 9.90)	0.837		
Multiple splenic infarction	0.91 (0.24, 3.44)	0.886		
Splenomegaly	0.04 (<0.001, 109.4)	0.424		
Anticoagulant therapy^†^	0.57 (0.13, 2.45)	0.446	0.71 (0.16, 3.09)	0.707

*HR, hazard ratio; 95% CI, 95% confidence interval*.

† *Anticoagulant therapy was analyzed as a time-dependent covariate by time-dependent Cox regression model*.

### Bleeding Events Assessment

Bleeding complications were evaluated as adverse events in patients with anticoagulation. Nine bleeding events occurred in the anticoagulation group (five due to gastrointestinal bleeding, one to non-traumatic intracranial hemorrhage, one to splenic rupture, one to hematuria, one to vaginal bleeding), while seven occurred in the nonanticoagulation group (five to gastrointestinal bleeding, one to traumatic intracranial hemorrhage, one to abdominal wound bleeding) during the follow-up period (Table 3). There was no statistically significant difference in the risk of bleeding events among patients who initiated anticoagulation compared to patients who did not receive anticoagulation (*p* = 0.728).

## Discussion

To the best of our knowledge, this is the first multicenter cohort study to evaluate the long-term outcomes of anticoagulant therapy in patients with SI. The strength of our study lies in the ED-based study design other than the results extracted from radiology databases. Although the diagnosis of SI is reported as an incidental finding in few patients, more than 80% of our patients presenting to the ED with symptoms of abdominal pain, back pain, left flank pain, and nausea or vomiting were attributed to SI. Another strength of our study is that almost all patients in the anticoagulation group were prescribed anticoagulants upon initial presentation in the ED, except two with anticoagulant therapy after admission to the general medical ward. These data describe the real-world utilization of anticoagulants in SI patients.

SI is a relatively uncommon diagnosis (10). Although more studies have been dedicated to determining the clinical characteristics, etiologies, and outcomes of SI in recent years, there are no studies that define the safety and efficacy of anticoagulant therapy using statistical methods in patients with SI. Antopolsky et al. (3) reported 48 patients diagnosed with SI in the ED. Long-term anticoagulation treatment was required in 17 patients with atrial fibrillation, hypercoagulable state, or SI itself, while the rest of the patients were treated for predisposing diseases or symptomatic treatment only. There was no in-hospital mortality. Schattner et al. (1) identified 32 patients with a diagnosis of SI, and all of them were given anticoagulants regardless of underlying etiologies. All reported patients survived to discharge, with the exception of one patient who had cardiogenic emboli to multiple sites. Bewersdorf et al. (7) conducted a large cohort study of SI in 206 cancer patients. The authors mentioned that there was no statistically significant difference in the risk of subsequent venous thromboembolism and recurrent SI among patients with or without anticoagulant therapy. However, in this study, the majority of SIs were asymptomatic and incidental findings on computed tomography (CT). Only 5.3% of patients reported left upper quadrant abdominal pain. Additionally, since the enrollment of this study was confined to only cancer patients from the radiology database, the outcomes of SI patients receiving anticoagulant therapy secondary to various etiologies in the acute setting remain unclear.

Our study suggests that anticoagulant therapy was independently associated with decreased long-term mortality. In our cohort, ischemic stroke comprised 30% of all mortalities in the non-anticoagulation group. The reason for the higher survival rate among the anticoagulation group is undetermined, but it might be the appropriate balance between antithrombotic and adverse bleeding effects. In a previously published series, thromboembolism, including cardiovascular etiologies and a hypercoagulable state, and a rapidly enlarging spleen, which indicates acute infection, malignancy, and hematologic disease, were two plausible causes responsible for SI (1, 3, 11, 12). Our study excluded patients with non-hematologic malignancy, hematologic disease, and infective endocarditis given that the different management strategies and prognoses of the underlying diseases increase the heterogeneity of the study population and might hinder the analysis of the impact of SI on overall survival. Thromboembolism constituted the predominant mechanism of SI in our patients. The common risk factors included a wide range of vascular diseases, atrial fibrillation, intracardiac thrombi, and autoimmune diseases. In addition, sepsis was frequently found in our patients with SI and was considered to be associated with the prothrombotic process (13–15). Bitzer et al. (16) reported a young woman with SI due to meningococcal septicemia. The patient underwent splenectomy, and histological examination of the resected spleen revealed large areas of disseminated thromboses in small- to medium-sized splenic veins. Both hypoperfusion due to septic shock and a hypercoagulable state contribute to an increased risk of SI. Notably, definite predisposing factors leading to SI could not be found in 20% of our patients. It is probable that unprovoked SIs are associated with non-documented thromboembolic diseases, such as silent atrial fibrillation. Indeed, cryptogenic stroke is a frequent consequence of underlying silent atrial fibrillation (17, 18). These data emphasize the importance of further investigations in SI patients without known predisposing diseases and the high potential of SI preventability with timely anticoagulant therapy.

Regarding thromboembolic events following a diagnosis of SI in our study, the Kaplan-Meier plot revealed a significantly higher event-free survival rate in patients with anticoagulant therapy than in those without treatment. However, after adjustment for the time-dependent nature of anticoagulant therapy exposure using a time-dependent Cox model, subsequent thromboembolic events did not significantly differ between the two groups. The reason for this result might be due to the small sample sizes and low event rates of this study, which was underpowered to detect a beneficial effect of anticoagulant therapy.

The major concerning adverse event related to anticoagulant therapy is the increased risk for intracranial hemorrhage and gastrointestinal bleeding (6, 19, 20). This study showed that there were no differences in bleeding event incidence between the two groups. Of note, as reflected in our study, splenic rupture may develop following an episode of infarction. Several reported cases described the association between anticoagulant therapy and subsequent splenic rupture (21–24). While ultrasound appears to have a low diagnostic yield for SI, it is useful in the detection of complications following SI, such as splenic rupture with hemoperitoneum (25, 26). A short-term follow-up with ultrasound is a reasonable strategy for patients in whom anticoagulants are initiated.

It is worth noting that a higher rate of underlying liver cirrhosis was found in patients without anticoagulant therapy. We suppose that clinicians prescribed fewer anticoagulants in these patients due to concerns about bleeding risk as a result of coagulopathy. Although cirrhotic patients have an increased risk of bleeding complications, they simultaneously present a pro-coagulant status that contributes to a higher incidence of thrombotic events (27, 28). Few reported cases have demonstrated the correlation between liver cirrhosis and SI (29, 30). The possible mechanisms are a prothrombotic state and increased oxygen demand from a congested spleen. Whereas we found no studies that confirmed anticoagulant use in cirrhotic patients with SI, anticoagulant therapy is recommended for symptomatic deep vein thrombosis, portal vein thrombosis, and splanchnic vein thrombosis (27, 31). Moreover, the treatment modality decreases the incidence of hepatic decompensation and improves overall survival (27).

Our study had several limitations. First, given its retrospective nature, this study did not allow us to accurately collect all clinical variables in a protocol form and was limited by missing data. In addition, there was possible selection bias and many confounding factors, although we attempted to perform adjustments using multivariate regression and time-dependent variables to minimize the risk of immortal time bias. Second, this study consisted of small numbers of patients and events that hampered our ability to draw clear-cut conclusions. For instance, while the observed thromboembolic event rate was lower in patients treated with anticoagulants than in those without treatment, this study was underpowered to detect a statistically significant difference between the two groups. Third, this study did not address the issues pertaining to the type, dose, and duration of anticoagulant therapy, as well as antiplatelet treatment. Further work is needed to clarify these important questions. However, it is also difficult to conduct a prospective comparative study among SI patients given the rarity of this disease.

## Conclusion

In summary, anticoagulant therapy was associated with decreased long-term mortality in SI patients. There was no significant difference in the subsequent bleeding events between the groups. Our findings emphasize the usefulness of anticoagulant therapy in SI patients and close monitoring of possible complications with better clinical outcomes.

## Data Availability Statement

The original contributions presented in the study are included in the article/supplementary material, further inquiries can be directed to the corresponding author.

## Author Contributions

C-CY: conceptualization, formal analysis, and writing—original draft. C-KW: data curation. J-PL and C-HC: investigation. S-YC: resources. C-JN: supervision. All authors have read and agreed to the published version of the manuscript.

## Conflict of Interest

The authors declare that the research was conducted in the absence of any commercial or financial relationships that could be construed as a potential conflict of interest.

## Publisher's Note

All claims expressed in this article are solely those of the authors and do not necessarily represent those of their affiliated organizations, or those of the publisher, the editors and the reviewers. Any product that may be evaluated in this article, or claim that may be made by its manufacturer, is not guaranteed or endorsed by the publisher.

## References

[1] Schattner A Adi M Kitroser E Klepfish A. Acute Splenic infarction at an academic general hospital over 10 years: presentation, etiology, and outcome. Medicine. (2015) 94:e1363. 10.1097/MD.0000000000001363 26356690 PMC4616622

[2] O'Keefe JH Jr Holmes DR Jr Schaff HV Sheedy PF 2nd Edwards WD. Thromboembolic splenic infarction. Mayo Clin Proc. (1986) 61:967–72. 10.1016/S0025-6196(12)62638-X 3773568

[3] Antopolsky M Hiller N Salameh S Goldshtein B Stalnikowicz R. Splenic infarction: 10 years of experience. Am J Emerg Med. (2009) 27:262–5. 10.1016/j.ajem.2008.02.014 19328367

[4] Lawrence YR Pokroy R Berlowitz D Aharoni D Hain D Breuer GS. Splenic infarction: an update on William Osler's observations. Isr Med Assoc J. (2010) 12:362–5. 20928991

[5] Maier W. Computed tomography in the diagnosis of splenic infarction. Eur J Radiol. (1982) 2:202–4. 7128603

[6] Piran S Khatib R Schulman S Majeed A Holbrook A Witt DM. Management of direct factor Xa inhibitor-related major bleeding with prothrombin complex concentrate: a meta-analysis. Blood Adv. (2019) 3:158–67. 10.1182/bloodadvances.2018024133 30658963 PMC6341194

[7] Bewersdorf JP Parmar N Israel GM Gettinger SN Lee AI. Clinical characteristics and outcomes of splenic infarction in cancer patients: a retrospective, single center report of 206 cases. J Thromb Thrombolysis. (2021). 10.1007/s11239-021-02428-0 33765243

[8] Jones M Fowler R. Immortal time bias in observational studies of time-to-event outcomes. J Crit Care. (2016) 36:195–9. 10.1016/j.jcrc.2016.07.017 27546771

[9] Bellera CA Macgrogan G Debled M De Lara CT Brouste V Mathoulin-Pélissier S. Variables with time-varying effects and the Cox model: some statistical concepts illustrated with a prognostic factor study in breast cancer. BMC Med Res Methodol. (2010) 10:20. 10.1186/1471-2288-10-20 20233435 PMC2846954

[10] Nores M Phillips EH Morgenstern L Hiatt JR. The clinical spectrum of splenic infarction. Am Surg. (1998) 64:182–8. 9486895

[11] Brett AS Azizzadeh N Miller EM Collins RJ Seegars MB Marcus MA. Assessment of clinical conditions associated with splenic infarction in adult patients. JAMA Intern Med. (2020) 180:1125–8. 10.1001/jamainternmed.2020.2168 32658244 PMC7358974

[12] Cox M Li Z Desai V Brown L Deshmukh S Roth CG. Acute nontraumatic splenic infarctions at a tertiary-care center: causes and predisposing factors in 123 patients. Emerg Radiol. (2016) 23:155–60. 10.1007/s10140-016-1376-3 26797023

[13] Giustozzi M Ehrlinder H Bongiovanni D Borovac JA Guerreiro RA Gasecka A. Coagulopathy and sepsis: pathophysiology, clinical manifestations and treatment. Blood Rev. (2021) 2021:100864. 10.1016/j.blre.2021.100864 34217531

[14] Dolmatova EV Wang K Mandavilli R Griendling KK. The effects of sepsis on endothelium and clinical implications. Cardiovasc Res. (2021) 117:60–73. 10.1093/cvr/cvaa070 32215570 PMC7810126

[15] Chen Z Zhang H Qu M Nan K Cao H Cata JP. Review: The emerging role of neutrophil extracellular traps in sepsis and sepsis-associated thrombosis. Front Cell Infect Microbiol. (2021) 11:653228. 10.3389/fcimb.2021.653228 33816356 PMC8010653

[16] Bitzer M Armeanu S Kröber SM Horger MS Erley CM. A young woman with splenic infarction. Lancet. (2003) 362:1456. 10.1016/S0140-6736(03)14691-0 14602441

[17] Dilaveris PE Kennedy HL. Silent atrial fibrillation: epidemiology, diagnosis, and clinical impact. Clin Cardiol. (2017) 40:413–8. 10.1002/clc.22667 28273368 PMC6490532

[18] Barbarossa A Guerra F Capucci A. Silent atrial fibrillation: a critical review. J Atr Fibrillation. (2014) 7:1138. 27957123 10.4022/jafib.1138 PMC4956292

[19] van den Ham HA Souverein PC Klungel OH Platt RW Ernst P Dell'Aniello S. Major bleeding in users of direct oral anticoagulants in atrial fibrillation: a pooled analysis of results from multiple population-based cohort studies. Pharmacoepidemiol Drug Saf. (2021) 30:1339–52. 10.1002/pds.5317 34173286 PMC8456818

[20] Menichelli D Del Sole F Di Rocco A Farcomeni A Vestri A Violi F. Real-world safety and efficacy of direct oral anticoagulants in atrial fibrillation: a systematic review and meta-analysis of 605 771 patients. Eur Heart J Cardiovasc Pharmacother. (2021) 7:f11–9. 10.1093/ehjcvp/pvab002 33493255

[21] Magnan H Kayton ML DiMichele DM Araten DJ Kernan NA Boulad F. Splenic infarction and subsequent splenic rupture in a patient with paroxysmal nocturnal hemoglobinuria and heparin-induced thrombocytopenia. Pediatr Blood Cancer. (2009) 53:472–4. 10.1002/pbc.22058 19415735

[22] Natarajan P Thangarasu S Ruck L Estrada P Gajendran M Renganathan G. Atraumatic splenic rupture in a patient on apixaban and dual antiplatelet therapy. J Investig Med High Impact Case Rep. (2021) 9:23247096211026492. 10.1177/23247096211026492 34148386 PMC8221677

[23] Díaz Alcázar MDM García Robles A Martín-Lagos Maldonado A. Splenic rupture as an endoscopic complication: as rare as it appears? Rev Esp Enferm Dig. (2021) 113:232. 10.17235/reed.2020.7342/2020 33222479

[24] Janke A Ikejiani S Mize C. Spontaneous splenic hemorrhage in a patient on apixiban. Am J Emerg Med. (2020) 38:1044.e1–.e2. 10.1016/j.ajem.2019.12.006 31932128

[25] Goerg C Schwerk WB. Splenic infarction: sonographic patterns, diagnosis, follow-up, and complications. Radiology. (1990) 174:803–7. 10.1148/radiology.174.3.2406785 2406785

[26] Gorg C Colle J Wied M Schwerk WB Zugmaier G. Spontaneous nontraumatic intrasplenic pseudoaneurysm: causes, sonographic diagnosis, and prognosis. J Clin Ultrasound. (2003) 31:129–34. 10.1002/jcu.10145 12594797

[27] O'Leary JG Greenberg CS Patton HM Caldwell SH. AGA clinical practice update: coagulation in cirrhosis. Gastroenterology. (2019) 157:34–43.e1. 10.1053/j.gastro.2019.03.070 30986390

[28] Ha NB Regal RE. Anticoagulation in patients with cirrhosis. Ann Pharmacother. (2016) 50:402–9. 10.1177/1060028016631760 26861989

[29] Erarslan E Bozkurt A Yüksel I Demir HD. Spontaneous splenic infarction in an elderly cirrhotic patient with multiple comorbidities. Turk J Gastroenterol. (2012) 23:596–8. 10.4318/tjg.2012.0429 23161308

[30] Mathieu B Le Gall P Arab K Mourani A Mora M Chevallier P. Massive splenic infarction in a patient with alcoholic cirrhosis. Gastroenterol Clin Biol. (2001) 25:1036–9. 11845063

[31] Valeriani E Riva N Di Nisio M Ageno W. Splanchnic vein thrombosis: current perspectives. Vasc Health Risk Manag. (2019) 15:449–61. 10.2147/VHRM.S197732 31695400 PMC6815215

